# Ensemble and temporal feature-based framework for rainfall classification in Bangladesh

**DOI:** 10.1371/journal.pone.0342646

**Published:** 2026-03-10

**Authors:** Mahir Shahriar Tamim, Md. Samiul Alim, Tanvir Ahmed Khan, Maisha Rahman, Md Musfique Anwar

**Affiliations:** 1 Department of Electrical and Computer Engineering, North South University, Dhaka, Bangladesh; 2 Department of Computer Science and Engineering, Jahangirnagar University, Dhaka, Bangladesh; UCSI University Kuala Lumpur Campus: UCSI University, MALAYSIA

## Abstract

Accurate rainfall classification is essential for Bangladesh, where monsoon variability strongly influences agriculture, water resource management, and disaster preparedness. This study proposes a robust machine learning framework for rainfall intensity classification at the **daily temporal scale** and **nationwide spatial coverage**, using over **543,839 daily weather records** collected from **35 meteorological stations** across several decades from a publicly available national meteorological dataset. The dataset includes rainfall, temperature, humidity, and sunshine duration, which were preprocessed and categorized into four intensity levels: No Rain, Light Rain, Moderate Rain, and Very Heavy Rain. Various models were evaluated, including Random Forest, Decision Trees, Gradient Boosting, K-Nearest Neighbors, Naïve Bayes, Extreme Gradient Boosting (XGBoost), Light Gradient Boosting Machine (LightGBM), and Categorical Boosting (CatBoost), along with deep learning architectures such as Artificial Neural Network (ANN), Deep Neural Network (DNN), One-Dimensional Convolutional Neural Network (1D-CNN), Long Short-Term Memory (LSTM), and Bidirectional LSTM (Bi-LSTM). Random Forest achieved the highest accuracy (77.37%), while Bi-LSTM performed best among deep learning models (76.97%). To address class imbalance, we adopted class weighting in the final models; SMOTE was explored as an ablation and then excluded due to poorer generalization. Model interpretability using Local Interpretable Model-Agnostic Explanations (LIME) and SHapley Additive exPlanations (SHAP) consistently identified humidity and sunshine as the most influential predictors, with SHAP further revealing strong interactions between lagged humidity and temperature. The framework‘s reliable classification of rainfall intensities supports data-driven irrigation scheduling, early flood warnings, and climate-resilient agricultural and disaster management planning in Bangladesh.

## Introduction

Rainfall classification remains a fundamental challenge in meteorology, particularly for monsoon-dependent nations like Bangladesh, where precipitation patterns govern agricultural productivity, water management, and disaster resilience [1,2]. The country‘s flat deltaic topography and extensive river networks exacerbate flood risks, while erratic rainfall induces drought cycles, collectively threatening food security and livelihoods [3,4]. Floods alone caused crop losses exceeding BDT 11.52 billion in 2019, underscoring the socio-economic urgency for accurate rainfall forecasting [5].

Traditional statistical and numerical weather prediction (NWP) models, such as ARIMA and regression-based frameworks, can struggle to represent strongly non-linear, multivariate, and time-varying dependencies in rainfall, particularly when relationships differ across seasons and regions [6,7]. These methods are often computationally demanding and may underperform in settings with complex spatiotemporal variability [8].

Recent advances in machine learning (ML) and deep learning (DL) have transformed rainfall forecasting by uncovering latent relationships among meteorological variables [1,3]. In Bangladesh, Random Forest, Gradient Boosting, and LSTM-based models have achieved promising accuracy, yet most studies remain limited to small, region-specific datasets or overlook interpretability [4,9]. Moreover, class imbalance where “No Rain” days dominate, often biases model predictions toward majority classes [10], reducing the reliability of extreme rainfall detection.

Our study advances rainfall classification in Bangladesh by leveraging a nationwide dataset of 543,839 records, far larger than previous regional studies [11,12], to benchmark ten machine learning models and five deep learning architectures, with Random Forest achieving the highest accuracy of 77.37%, outperforming prior approaches [13,1]. We introduce lag-based and cyclical temporal features, improving accuracy by approximately 4%, and employ a Greedy Ensemble Selection that attains 74% accuracy for extreme events. Post-hoc interpretability using LIME highlights humidity and sunshine as key predictors. To handle class imbalance, we adopt class weighting in all final reported models, while SMOTE was evaluated as an ablation and excluded due to poorer generalization [14]. Unlike classical statistical models such as LDA and QDA, which rely on strong distributional and decision-boundary assumptions, our framework models non-linear and multivariate relationships present in meteorological data, offering enhanced predictive performance, interpretability, and generalizability critical for actionable, policy oriented climate forecasting.

## Related work

Rainfall classification and prediction research has evolved from linear statistical methods to modern ML and DL paradigms, reflecting a shift from parametric modeling toward data-driven discovery of complex atmospheric dependencies. This section outlines that progression and highlights persistent gaps motivating our proposed approach.

**Traditional and Statistical Models:** Early approaches such as ARIMA, fuzzy time series, and trend-detection methods (e.g., MAKESENS, Pettitt) provided interpretable baselines but were inadequate for capturing rainfall‘s stochastic variability [15]. Olatayo and Taiwo [15] reported 15–20% RMSE reductions with fuzzy systems over ARIMA, yet both suffered from rigid parametric assumptions. In Bangladesh, Monir et al. [13] combined MAKESENS with MLP for 28 stations (1981–2020), offering decadal projections but with limited accuracy due to breakpoint misdetection. While computationally efficient, these models fail to generalize across regions or accommodate multivariate dependencies [14].

**Machine Learning Methods:** ML models such as Random Forest (RF), Support Vector Machines (SVM), Decision Trees (DT), and Gradient Boosting have demonstrated the capacity to model non-linear interactions among climatic variables, achieving 80–90% accuracy in controlled datasets. In Bangladesh, Ria et al. [11] achieved an F1-score of 0.85 with RF but encountered overfitting from sparse features, while Ahammad et al. [12] attained F1 0.82 using Gradient Boosting–Logistic Regression hybrids, noting humidity-related misclassifications. Rajab et al. [1] employed KNN–SVM–ANN models for flood forecasting but achieved limited recall (75%) on extreme events. Globally, RF and ensemble models have shown robustness [17,16], yet they remain sensitive to class imbalance and struggle to capture temporal correlations [18].

**Deep Learning and Hybrid Architectures:** DL models LSTM, Bi-LSTM, GRU, and CNN extend ML capabilities by learning sequential dependencies in rainfall time series, with typical accuracies between 75–85% [20,19]. Tanzim and Yasmin [19] used an SVM–CNN hybrid for Bangladesh thunderstorms, achieving F1 0.82 but with high computational overhead. Aswin et al. [20] and Wani et al. [20] demonstrated that LSTM-based models outperform ML baselines, though generalization declines by 10–15% in cross-regional testing. Hybrid ML–DL combinations, such as DT–DL [21] or PCA–LSTM [22], have improved non-linear representation but still exhibit poor interpretability and degraded performance for minority rainfall classes [23].

**Synthesis and Research Gaps:** Systematic reviews reveal consistent limitations: dataset heterogeneity, inconsistent normalization, inadequate temporal modeling, and lack of interpretability [14,24]. In Bangladesh, most studies use regional datasets (typically < 10,000 samples), omit nationwide coverage, and disregard the 68% dominance of “No Rain” days, resulting in biased forecasts [18,25,26,27]. Furthermore, few studies integrate post-hoc interpretability or ensemble optimization into rainfall classification frameworks.

This study fills these gaps by leveraging a nationwide dataset of 543,839 records across 35 stations, employing advanced temporal feature engineering, Greedy Ensemble Selection, and LIME-based interpretability. Our approach balances predictive accuracy, fairness, and transparency critical factors for operational integration in climate-resilient policy planning.

Representative prior studies and their limitations are summarized in Table 1.

**Table 1:** Recent approaches to rainfall prediction across various methodological frameworks.

Reference	Contribution	Limitations
Nushrat Jahan Ria et al. (2023) [11]	Applied multiple ML models on a Bangladesh-specific dataset, identifying RF as most accurate.	Dataset limited to 2,391 records; lacks feature details.
Md. Saymon Ahammad et al. (2023) [12]	Used hybrid models on station-wise data from BRRI (2016–2020).	Model performance affected by data balancing challenges.
Md. Moniruzzaman Monir et al. (2023) [13]	Combined MLP with MAKESENS and IAA for trend analysis.	Unable to detect exact break years in series.
Adel Rajab et al. (2023) [1]	Used historical BMD data for rainfall/flood prediction with ML.	Scope limited; few deep learning models tested.
Suman Markuna et al. (2022) [16]	Assessed ML models (RF, MARS, SVR, MLR) on Himalayan data.	Poor simulation of extreme rainfall events.
Mahiyat Tanzim and Sabina Yasmin (2025) [19]	Used CNN and SVM for thunderstorm classification in Bangladesh.	CNN requires high computation and faces generalization issues.
Aswin S et al. (2021) [20]	Compared ConvNet and LSTM on GPCP global data.	Minor overfitting; external factors not assessed.
Jamal Hussain et al. (2022) [24]	Reviewed hybrid ML models across studies.	No evaluation of hybrid DL-physics models.
B. Meena Preethi et al. (2022) [21]	Developed DT-based hybrid DL model for rainfall.	Dataset unspecified; overfitting concerns.
Owais Ali Wani et al. (2023) [20]	Compared ML, DL, and ARIMA across altitudinal regions.	Data sparsity in high-altitude stations.
T.O. Olatayo and A.I. Taiwo (2021) [15]	Compared Fuzzy Time Series, ARIMA, Theil‘s regression.	ARIMA assumption issues; fuzzy outperformed.
Tharun V.P et al. (2021) [17]	Used SVR, RF, DT on Tamil Nadu station data (2005–2014).	High atmospheric variability impacts accuracy.
Jamal Hussain and Zoremsanga (2022) [14]	Reviewed DL using satellite, radar, and station data.	Missing benchmarks; no hybrid evaluations.

## Materials and methods

### Overview of the proposed framework

**Fig 1:**
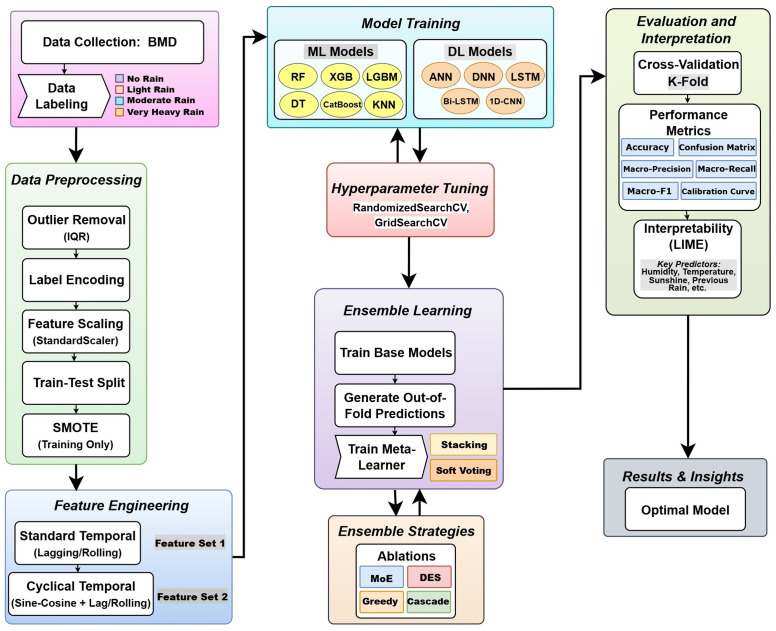
Workflow of the proposed pipeline. The diagram outlines the sequential stages of data collection, preprocessing, feature engineering, model training, evaluation, and result interpretation.

The overall workflow of the proposed rainfall classification framework, from data collection and preprocessing to feature engineering, model training, ensembling, and interpretability, is shown in Fig 1.

### Dataset

**Fig 2:**
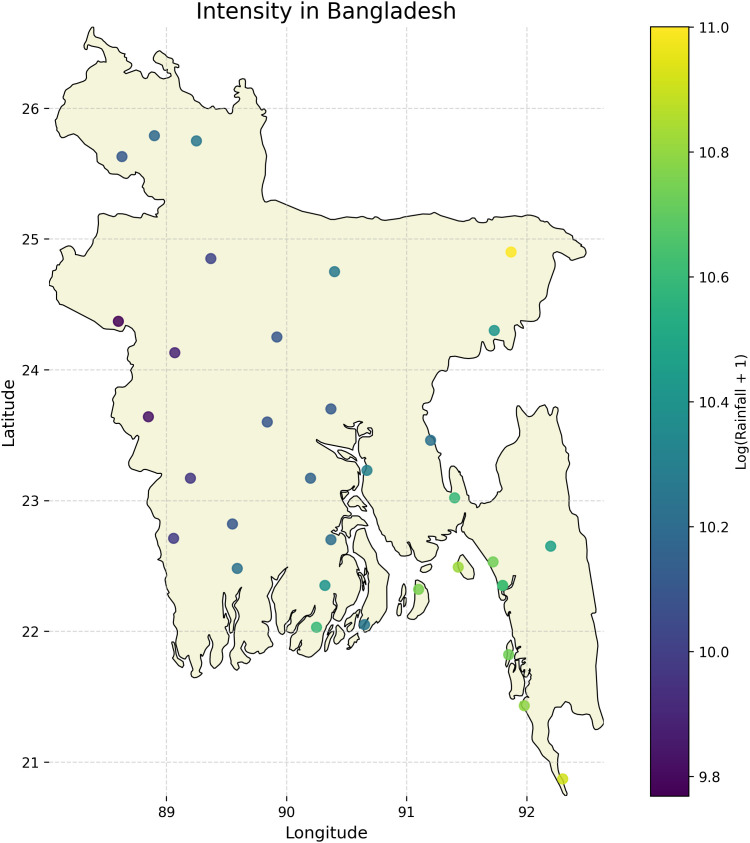
Rainfall intensity distribution across Bangladesh. The figure visualizes cumulative precipitation from 2010 onward. The map highlights regional variations in rainfall patterns, offering insights into long-term hydrological trends. *Base map data from Natural Earth (public domain), with additional layers and annotations by the authors*.

The dataset described by Zubair et al. [28] offers a comprehensive compilation of daily weather data from 35 meteorological stations across Bangladesh, spanning several decades and encompassing approximately 543,839 rows. It covers four key atmospheric parameters: rainfall (mm), temperature (C), humidity (%), and sunshine duration (hours). Collected by the Bangladesh Meteorological Department (BMD), this high-volume dataset underwent rigorous preprocessing to address inconsistencies, missing values, and formatting issues. The result is a structured, machine-learning-ready resource suitable for diverse applications such as climate modeling, agricultural planning, and public health research, as highlighted in the original study. Fig 2 presents a map visualization illustrating the cumulative rainfall intensity across Bangladesh since 2010.

To enhance its utility for classification tasks, we introduced an additional feature named Rainfall_Class, derived from the original continuous rainfall measurements. This transformation categorized rainfall values into four discrete classes based on simplified meteorological thresholds, following the classification approach outlined by Baten et al. [29]:

- **Class 0 (No Rain):** Rainfal = 0.0 mm
- **Class 1 (Light Rain):** 1–10 mm
- **Class 2 (Moderate Rain):** 11–43 mm
- **Class 3 (Very Heavy Rain):** ≥44 mm

**Fig 3:**
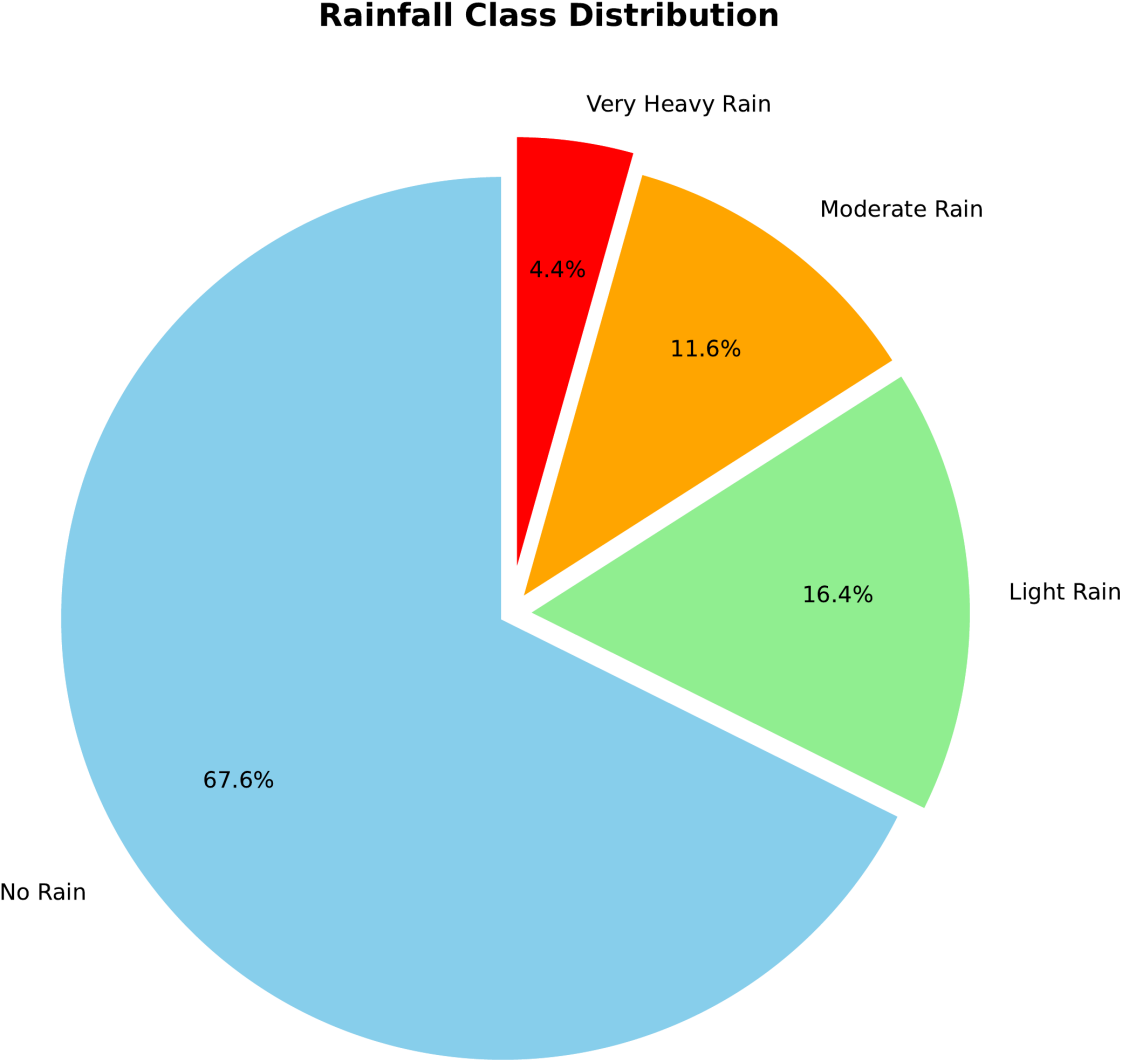
Dataset class distribution. The figure illustrates the frequency of samples across the four rainfall intensity classes (Class 0–3), highlighting the class imbalance in the dataset.

As shown in the Rainfall Class Distribution in Fig 3, the dataset exhibits a noticeable class imbalance. Class 0 (No Rain) dominates with 367,879 instances, constituting approximately 68% of the total samples. In contrast, Class 3 (Very Heavy Rain) is significantly underrepresented, with only 23,764 instances (roughly 4%). This distribution closely mirrors real-world meteorological patterns in Bangladesh, where dry days are far more frequent than instances of extreme rainfall.

This classification facilitates modeling tasks where categorical outputs are preferred, such as in disaster preparedness, crop yield forecasting, and educational platforms. To streamline the dataset and eliminate redundancy, the original Rainfall column was removed after the creation of Rainfall_Class.

**Justification for classification over regression.** Although regression-based rainfall estimation (predicting continuous mm values) is a valid alternative, we adopted a classification framework for several reasons. First, operational decision-making in flood early warning, irrigation scheduling, and disaster preparedness is inherently threshold-based: stakeholders act on categorical alerts (e.g., “heavy rain expected”) rather than precise millimeter forecasts [1,3]. Classification directly produces actionable intensity categories aligned with Bangladesh Meteorological Department (BMD) warning protocols. Second, rainfall distributions are highly skewed and zero-inflated (68% of days have no rain), which violates the homoscedasticity and normality assumptions underlying many regression models and complicates error interpretation [30]. Third, classification enables the use of well-established metrics (precision, recall, F1-score) that transparently quantify performance on rare but high-impact events (e.g., Very Heavy Rain), whereas regression metrics such as RMSE can be dominated by abundant low-rainfall days. Finally, probabilistic classifiers output confidence scores that directly support the reliability-aware decision framework developed in this study, enabling selective automation of high-confidence predictions. For these application-driven reasons, classification was deemed more suitable than regression for this operational rainfall forecasting task.

### Exploratory data analysis

**Fig 4:**
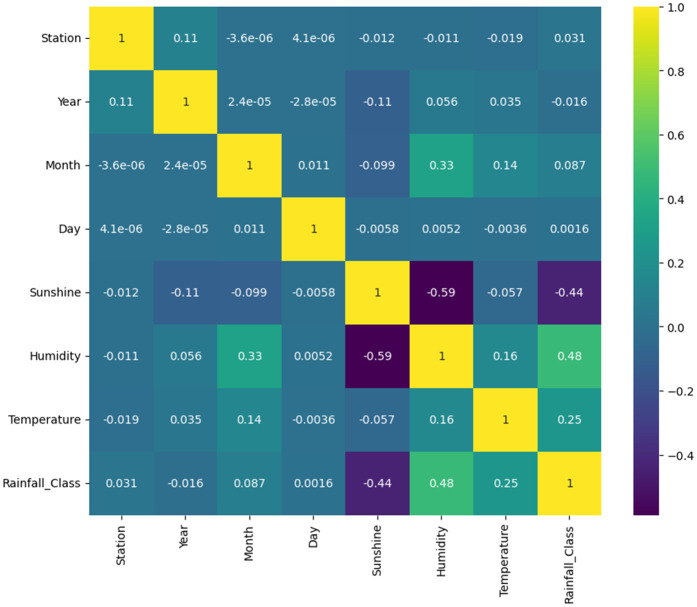
Correlation matrix of meteorological variables. The figure depicts pairwise Pearson correlation coefficients among the principal meteorological variables—temperature, humidity, and sunshine duration—and the target variable, rainfall class. Positive correlations are indicated by warmer tones and negative correlations by cooler tones. The analysis reveals humidity as the most positively correlated predictor and sunshine duration as the most negatively correlated factor influencing rainfall intensity across Bangladesh.

The correlation matrix (Fig 4) summarizes pairwise linear relationships among predictors and the target variable (**Rainfall_Class**). The strongest positive linear association with rainfall class is **humidity** (Pearson r≈0.48 ), while **sunshine** exhibits a moderate negative correlation (r≈−0.44 ). **Temperature** shows a weak positive relationship (r≈0.25 ); station and month display negligible linear correlations with the target (station r≈0.03 , month r≈0.09 ). These results indicate that humidity and sunshine duration are the primary linear drivers of class separation in this dataset, whereas temperature provides a secondary signal. We note, however, that weak linear correlations for station and month do not preclude important non-linear or interaction effects; therefore, our modelling and feature-engineering strategy explicitly targets both non-linearity and temporal cyclicity. Summary statistics for the main variables are reported in Table 2.

**Table 2:** Descriptive statistics for the main variables.

Feature	Mean	Std	Min	Q1	Median	Q3	Max
Rainfall (mm)	6.73	19.37	0.0	0.0	0.0	3.0	590.0
Temperature (°C)	25.57	4.17	0.0	22.6	26.9	28.8	37.8
Humidity (%)	79.85	8.94	10.0	75.0	81.0	86.0	100.0
Sunshine (hrs)	6.28	3.35	0.0	3.8	7.2	9.0	90.2

### Spatial distribution of weather parameters

**Fig 5:**
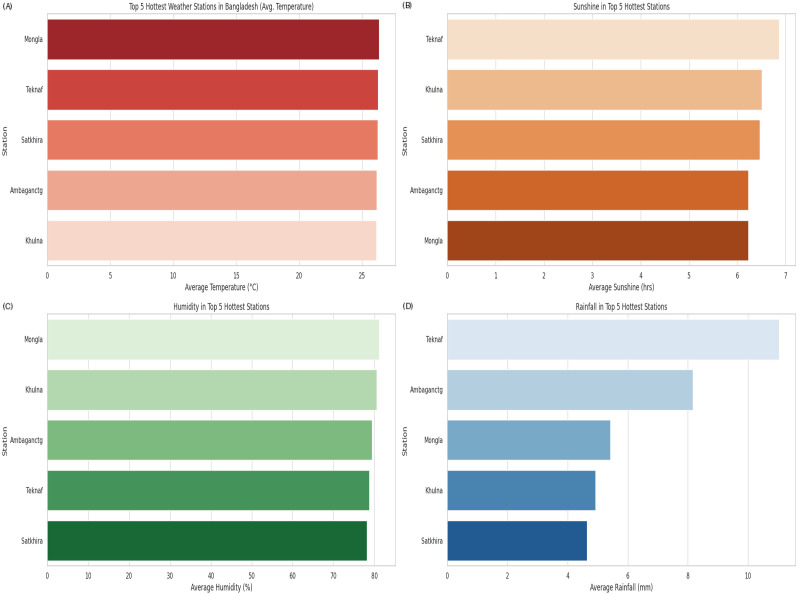
Regional weather variations across Bangladesh’s five hottest stations. (A) Average temperature, (B) sunshine duration, (C) relative humidity, and (D) rainfall patterns. Data reflect annual averages from the study period.

We analyzed station-level variability in four key meteorological variables across the five hottest stations in Bangladesh. The corresponding visualizations are presented in Fig 5 (panels A–D).

As shown in Fig 5A, Mongla, Teknaf, and Satkhira exhibit the highest mean temperatures, consistent with coastal and southwestern climatic influences. These patterns emphasize the importance of incorporating regional thermal variability into station-wise modeling.

Fig 5B presents sunshine duration, with Teknaf showing the highest annual values. This aligns with the inverse sunshine–rainfall relationship identified in the correlation analysis, suggesting that spatial differences in cloud cover are relevant for rainfall classification.

Relative humidity patterns in Fig 5C indicate elevated moisture levels (70–80%) in Mongla and Khulna regions strongly affected by Bay of Bengal moisture transport. Given humidity‘s strong predictive value, these stations contribute significantly to rainfall signal dynamics.

Rainfall distributions in Fig 5D show that Teknaf receives the most rainfall (around 10 mm), while Satkhira and Khulna receive substantially less (4–6 mm). This highlights pronounced spatial heterogeneity in precipitation regimes across the country.

Overall, the spatial analysis demonstrates consistent meteorological relationships particularly the dominant roles of humidity and sunshine while underscoring the need for location aware modeling in the proposed classification framework.

### Data preprocessing

Several preprocessing steps were applied to ensure high-quality inputs for the machine learning models, including outlier removal, categorical encoding, feature scaling, class-imbalance handling, and a train–test split. These steps enhance model stability, accuracy, and generalization.

**Outlier removal using the IQR method.** Outliers can distort statistical patterns and degrade model performance. We therefore used the Interquartile Range (IQR) method to identify and remove extreme values from numerical variables (e.g., temperature, humidity).

*Quartile computation:*


$$Q_{1}=\text{25th percentile},\;Q_{3}=\text{75th percentile},\;$$

$$IQR=Q_{3}-Q_{1}.\;$$

*Outlier bounds:*


$$\text{Lower Bound}=Q_{1}-1.5\times IQR,\;\text{Upper Bound}=Q_{3}+1.5\times IQR.\;$$

*Filtering rule:* Data were retained only if they satisfied


Lower Bound≤x≤Upper Bound. 

All values outside this interval were considered outliers and removed.

**Label encoding for categorical features.** The dataset includes categorical entries (e.g., Station). Since many machine learning algorithms require numerical features, each unique station name was converted to a distinct integer label. This preserved station identity while ensuring algorithmic compatibility.

**Feature scaling using StandardScaler.** To normalize numerical variables and ensure equal contribution during training, features were standardized to zero mean and unit variance using the transformation in Eq. (1) [31].


$$z=\frac{x-\mu }{\sigma },\;$$ (1)

where *x* is the original value, *μ* is the feature mean, and *σ* is the standard deviation.

**Train–test split.** Following preprocessing, the dataset was divided into:

- 80% for training, and
- 20% for testing.

This ensured unbiased evaluation on unseen data and prevented information leakage between training and testing phases.

**Handling class imbalance (class weighting; SMOTE ablation).** Rainfall classes are highly imbalanced, with *No Rain* dominating the distribution. To mitigate this in the final modelling framework, we used class weighting during training. In addition, we evaluated the Synthetic Minority Over-sampling Technique (SMOTE) as an ablation on the training set only; for completeness, SMOTE is summarized below and formalized in Eq. (2).

*SMOTE algorithm formulation [10]:*

Given a minority-class instance $x_{i}\;$ and one of its *k* nearest neighbors $x_{zi}\;$, SMOTE generates:


$$x_{\text{new}}=x_{i}+\delta (x_{zi}-x_{i}),\;$$ (2)

where δ∈[0,1]  is a random scalar.

This increases minority-class density without simple duplication and helps balance class representation during training.

*Protocol note.* Although SMOTE was tested as an ablation, it significantly reduced performance (e.g., Random Forest accuracy decreased from ∼0.73–0.74 to 0.38). Therefore, all final reported models excluded SMOTE and instead used class weighting and temporally informed features.

### Local Interpretable Model-Agnostic Explanations

Local Interpretable Model-Agnostic Explanations (LIME) is a post-hoc explainability technique that interprets individual predictions of black-box models by approximating them locally with interpretable surrogate models (linear models or decision trees) [32].

Given an instance x , LIME generates perturbed samples $x^{\prime }\;$ by randomly altering feature values. Each perturbed instance is then assigned a weight based on its proximity to x , using a kernel function as shown in Equation 3 [32].


$$\pi_{x}(x^{\prime })=\exp\left(-\frac{D(x,x^{\prime }{)}^{2}}{\sigma^{2}}\right)\;$$ (3)

where D(·)  is a distance metric (e.g., cosine similarity or Euclidean distance), and σ  controls the width of the locality.

A simple surrogate model g∈G  is trained on the weighted dataset to approximate the complex model f  locally, by minimizing the following objective (Equation 4) [32]:


$$\xi (x)=\arg\min_{g\in G}\sum_{i}\pi_{x}(x_{i}^{\prime }){\left(f(x_{i}^{\prime })-g(x_{i}^{\prime })\right)}^{2}+\Omega (g)\;$$ (4)

Here, Ω(g)  is a complexity penalty term that enforces interpretability (e.g., sparsity in linear models). The learned coefficients of g  highlight the most influential features for the prediction f(x) .

We applied LIME to interpret rainfall class predictions (Class 0–3) in our preprocessed dataset. For example, to explain why a specific day was classified as Class 3 (Very Heavy Rain), LIME generates perturbations of the input features and trains a local surrogate model.

### SHapley Additive exPlanations (SHAP)

SHapley Additive exPlanations (SHAP) is a unified framework for interpreting model predictions based on principles from cooperative game theory [33]. SHAP assigns each feature an importance value that reflects its contribution to the prediction, ensuring a fair and theoretically grounded distribution of the model output among all input features.

For a given prediction instance x , the model f(x)  is decomposed into an additive explanation model as shown in Equation 5:


$$f(x)=\phi_{0}+\sum_{i=1}^{M}\phi_{i},\;$$ (5)

where $\phi_{0}\;$ is the expected model output (baseline prediction over all samples), M  is the number of features, and $\phi_{i}\;$ represents the Shapley value – i.e., the marginal contribution of feature i  to the difference between f(x)  and the baseline.

The Shapley value for feature i  is formally defined as:


$$\phi_{i}=\sum_{S\subseteq F⧵\{i\}}\frac{|S|!\;(M-|S|-1)!}{M!}\left[f_{S\cup \{i\}}(x_{S\cup \{i\}})-f_{S}(x_{S})\right],\;$$ (6)

where F  denotes the set of all features, and $f_{S}(x_{S})\;$ is the model prediction using only the subset of features S . The weighting term ensures that contributions are averaged over all possible feature orderings, yielding a unique and consistent attribution for each feature.

SHAP values satisfy three desirable properties: **local accuracy**, ensuring that the sum of feature contributions equals the model output; **missingness**, ensuring that features absent in a model have zero contribution; and **consistency**, meaning that feature importance does not decrease when its contribution to the model increases.

In this study, SHAP was applied to interpret both ensemble and deep learning model predictions for rainfall intensity classification. Global SHAP summary plots were used to identify the most influential features across the dataset, while dependence plots revealed nonlinear interactions, particularly between lagged humidity and temperature, providing physically consistent insights into rainfall behavior across Bangladesh.

### Monthly variations in weather parameters

**Fig 6:**
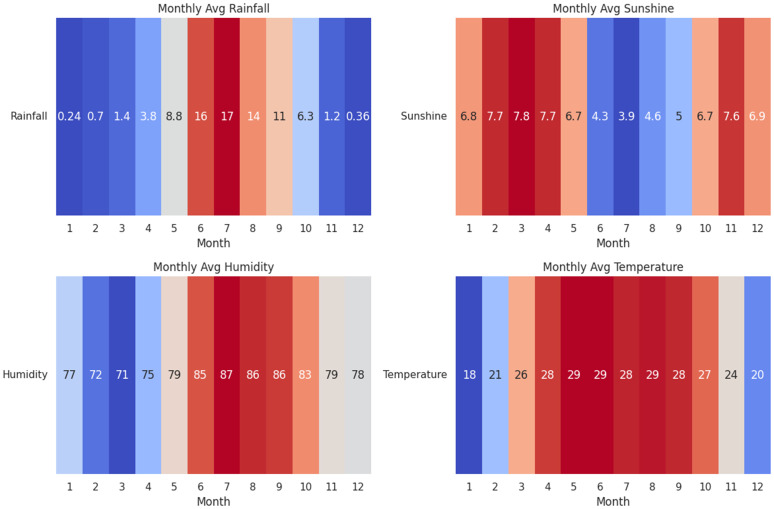
Monthly variations of weather parameters in Bangladesh. The figure shows: (a) average rainfall (mm), (b) sunshine duration (hours), (c) relative humidity (%), and (d) temperature (°C) across a calendar year. The data highlight the characteristic monsoon pattern, with peak rainfall occurring from June to August and an inverse relationship with sunshine hours. Humidity remains persistently high (>70%) throughout the year, while temperature exhibits expected seasonal variation, with the highest values in the pre-monsoon months (April–June).

The diagram in Fig 6 illustrates clear seasonal trends across Bangladesh’s key weather variables. Rainfall shows a pronounced monsoon pattern, with the heaviest precipitation occurring from June to August (16–17 mm monthly average) and a secondary peak in April–May (3.6–8.8 mm), while January–February and November–December remain driest (< 1 mm). Sunshine hours exhibit an inverse relationship with rainfall, reaching their highest duration during the pre-monsoon months of March–April (7.8 hours) and dropping significantly during the monsoon season (June–July: ∼4 hours) due to persistent cloud cover.

Humidity levels remain consistently high throughout the year (>70%) but peak during July–September (86–87%), reflecting saturated atmospheric conditions during peak monsoon. Temperature variations follow expected seasonal patterns, with the hottest conditions in April–June (28–29^°^C) and coolest temperatures in January (18^°^C) and December (20^°^C).

These patterns collectively characterize Bangladesh’s tropical monsoon climate, highlighting the strong interdependence between humidity, sunshine, and rainfall intensity. The bimodal rainfall distribution and inverse sunshine–rainfall relationship are particularly noteworthy for agricultural and water resource applications.

### Feature Engineering

To effectively capture temporal dependencies and periodic behaviors within the weather data, two distinct feature sets were developed: **Feature Set 1 (Standard Time Features)** and **Feature Set 2 (Cyclical Time Features)**. Both sets incorporate lag and rolling window operations but differ in their treatment of time-based attributes and encoding of periodicity.

#### Feature Set 1: Standard Time Features.

Feature Set 1 aims to represent temporal dependencies through explicit calendar-based attributes, lagged variables, and rolling statistical measures. From the timestamp variable (ds ), standard temporal components such as month (m=ds.month ), day of the week ($d_{w}=\text{ds.dayofweek }$), and day of the year ($d_{y}=\text{ds.dayofyear }$) were extracted to capture seasonal and weekly variations.

To model short-term temporal dependencies, lag features were generated for key meteorological variables, including temperature, humidity, and sunshine, at intervals of 1, 3, and 7 days [30]. For a given variable *f* and lag interval *l*, each lag feature was defined as shown in Equation 7:


$$f_{{\text{lag}}_{l}}=f_{t-l},\;$$ (7)

where $f_{t-l}\;$ represents the value of the variable *f* observed *l* days earlier for the same station.

In addition, rolling statistical descriptors were computed using a seven-day moving window to capture short-term variability and smoothing effects [34]. The moving average and standard deviation were calculated using Equations 8 and 9, respectively:


$$f_{{\text{roll\_mean}}_{7}}=\frac{1}{7}\sum_{i=0}^{6}f_{t-i},\;$$ (8)

$$f_{{\text{roll\_std}}_{7}}=\sqrt{\frac{1}{7}\sum_{i=0}^{6}(f_{t-i}-f_{{\text{roll\_mean}}_{7}}{)}^{2}}.\;$$ (9)

Equations 8 and 9 quantify the local temporal dynamics by describing short-term trends and volatility in the meteorological variables. Missing values were treated per station (unique_id ) using forward fill, backward fill, and zero-fill techniques to ensure completeness.

The selection of lag intervals (1, 3, and 7 days) and the 7-day rolling window is grounded in established atmospheric dynamics relevant to Bangladesh’s monsoon climate. The 1-day lag captures immediate persistence effects arising from synoptic-scale pressure systems that typically persist for 24–48 hours. The 3-day lag corresponds to the characteristic passage time of weather disturbances and frontal systems during pre-monsoon and post-monsoon transition periods. The 7-day lag and rolling window capture weekly-scale variability associated with intraseasonal monsoon fluctuations and the Madden-Julian Oscillation (MJO), which modulates rainfall over South Asia on 7–14 day timescales. These meteorologically motivated intervals ensure that the most physically relevant temporal dependencies are captured while maintaining computational efficiency.

**Fig 7:**
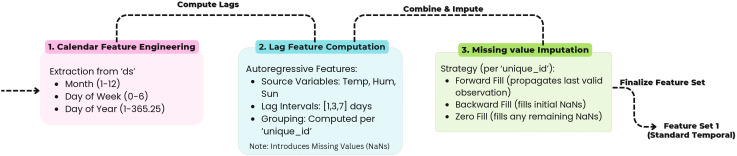
Feature engineering pipeline for Feature Set 1 (standard temporal encoding). The diagram outlines the sequential steps of temporal feature extraction, including calendar-based attributes, lag computation, and rolling window aggregation.

The complete workflow of Feature Set 1 is depicted in Fig 7, which illustrates the sequential stages of calendar attribute extraction, lag computation, and rolling feature aggregation.

#### Feature Set 2: Cyclical Time Features.

Feature Set 2 extends the temporal representation by incorporating cyclical encodings to better model periodic variations such as annual and monthly cycles. Following Chakraborty et al. [35] and NVIDIA [36], cyclic transformations were applied to time variables using sine and cosine functions. This approach prevents discontinuities at the boundaries of periodic attributes (e.g., December to January) and facilitates smoother learning of seasonal patterns. The transformations are defined in Equations 10 and 11:


$$d_{y}^{\sin}=\sin\left(2\pi \cdot \frac{\text{ds.dayofyear}}{365.25}\right),\;d_{y}^{\cos}=\cos\left(2\pi \cdot \frac{\text{ds.dayofyear}}{365.25}\right),\;$$ (10)

$$m^{\sin}=\sin\left(2\pi \cdot \frac{\text{ds.month}}{12}\right),\;m^{\cos}=\cos\left(2\pi \cdot \frac{\text{ds.month}}{12}\right),\;$$ (11)

where $d_{y}^{\sin}\;$ and $d_{y}^{\cos}\;$ (Equation 10) capture annual periodicity, while $m^{\sin}\;$ and $m^{\cos}\;$ (Equation 11) encode intra-annual cycles. Lag and rolling features, defined in Equations 7–9, were similarly applied to preserve short-term temporal dependencies within this cyclical encoding framework.

Together, these engineered features substantially enhanced model performance across both traditional and deep learning algorithms. In particular, Random Forest and LightGBM achieved test accuracies exceeding 77%, confirming the effectiveness of temporal and cyclical encodings in capturing rainfall variability across Bangladesh.

### Machine learning models

To benchmark rainfall classification, we selected numerous machine learning algorithms spanning linear, probabilistic, non-parametric, and ensemble families. This diversity ensured coverage of both simple interpretable models and high-capacity non-linear learners well suited to meteorological data.

Among linear classifiers, Logistic Regression (LR) served as a fundamental baseline for rainfall intensity classification. LR estimates the probability of class membership using a linear decision boundary and is valued for its computational efficiency, interpretability, and well-understood statistical properties. In the context of this study, LR provides a reference point against which non-linear models can be compared, helping to quantify the performance gains attributable to more complex algorithms. However, the principal limitation of LR is its inability to capture higher-order feature interactions—a significant drawback for meteorological datasets where predictors such as humidity, temperature, and sunshine duration exhibit complex interdependencies that influence rainfall outcomes.

To represent probabilistic modeling approaches, Gaussian Naive Bayes (GNB) was included as a generative classifier. GNB operates under the assumption of conditional feature independence given the class label, along with Gaussian-distributed continuous inputs, which substantially simplifies computation and enables rapid training even on large datasets. Although these assumptions rarely hold exactly in meteorological data—where humidity and temperature are often correlated—GNB nonetheless provides a useful probabilistic baseline and performs reasonably well on imbalanced classification tasks due to its ability to estimate class-conditional densities. The primary disadvantage of GNB is its reduced flexibility when predictors violate the independence assumption, potentially leading to suboptimal decision boundaries.

As a representative of non-parametric methods, K-Nearest Neighbors (KNN) was evaluated to assess instance-based learning on rainfall classification. KNN determines predictions through majority voting among the *k* nearest training samples in feature space, making it highly flexible and capable of capturing arbitrarily complex class boundaries without imposing parametric assumptions. This flexibility is advantageous for meteorological data where class boundaries may be irregular. However, KNN suffers from well-known limitations: sensitivity to irrelevant or redundant features, degraded performance without proper feature scaling, and high computational cost during inference on large datasets such as ours (543,839 samples). Despite these drawbacks, KNN was included to provide a comprehensive comparison of algorithmic families.

Tree-based methods constitute the core of our model selection due to their proven effectiveness on structured tabular data. Decision Tree (DT) classifiers recursively partition the feature space by selecting splits that maximize information gain or minimize impurity, producing transparent and interpretable models. However, single DTs are prone to overfitting, particularly on high-dimensional feature sets. Random Forest (RF) addresses this limitation by constructing an ensemble of DTs trained on bootstrapped samples with random feature subsets at each split, thereby reducing variance through bagging and improving generalization. RF has demonstrated strong performance across diverse meteorological prediction tasks and handles mixed feature types effectively. Gradient Boosting Machine (GBM) and AdaBoost adopt an alternative ensemble strategy, constructing trees sequentially where each subsequent tree is trained to correct the residual errors of the cumulative ensemble. This boosting approach captures subtle non-linear patterns in rainfall data but requires careful regularization to avoid overfitting on noisy features.

Building upon classical gradient boosting, we evaluated three modern boosting frameworks—XGBoost, LightGBM, and CatBoost—that have achieved state-of-the-art results on structured data benchmarks. XGBoost extends gradient boosting with L1 and L2 regularization terms and employs efficient parallelization strategies, making it both powerful and scalable to large datasets. LightGBM further improves training efficiency through histogram-based gradient computation and leaf-wise (rather than level-wise) tree growth, which accelerates convergence and provides better handling of class imbalance through built-in weighting mechanisms. CatBoost introduces ordered boosting to reduce prediction shift and employs specialized target encoding for categorical features, yielding models that are robust and less prone to overfitting without extensive hyperparameter tuning. These advanced boosting methods are particularly well-suited to our rainfall classification task, as they balance computational efficiency with high predictive accuracy while providing robustness in the presence of the highly skewed class distribution (68% “No Rain” samples) characteristic of Bangladesh’s climate.

Across these classical model families, decision boundaries are learned through different aggregation strategies: single decision trees form piecewise-constant partitions by greedily selecting impurity-reducing splits; Random Forest reduces variance via bagging (bootstrapped trees with random feature subsetting); and boosting builds an additive ensemble sequentially, where each new learner is fit to reduce the residual error of the current ensemble. Similar model families paired with SHAP-based post-hoc interpretation have been used to explain feature contributions in related applied prediction tasks (e.g., [37]).

These machine learning models serve as interpretable and computationally efficient baselines, providing performance benchmarks against which the deep learning architectures can be evaluated.

In contrast to classical statistical classifiers such as Linear Discriminant Analysis (LDA), Quadratic Discriminant Analysis (QDA), and related generative models, the models used in this study are designed to learn non-linear and multivariate relationships among predictors. LDA/QDA rely on assumptions (e.g., Gaussian class-conditional distributions and linear/quadratic decision boundaries) that may not hold for our heterogeneous feature set with temporal lags and cyclical encodings. Given our objective of robust nationwide rainfall intensity classification, we prioritized ensemble and non-parametric algorithms (e.g., Random Forest, Gradient Boosting, and XGBoost) that are less restrictive in their assumptions and empirically perform well on structured meteorological data.

### Hyperparameter tuning

To improve the predictive performance of the models used for rainfall classification, rigorous hyperparameter optimization was performed. The goal was to identify the best configuration of hyperparameters for each algorithm, thereby maximizing their classification accuracy on the dataset.

#### Search strategy.

Two complementary search techniques were employed to explore the hyperparameter spaces: RandomizedSearchCV and GridSearchCV, both from the scikit-learn framework. These methods enabled systematic and efficient tuning of model parameters.

RandomizedSearchCV samples a fixed number of hyperparameter combinations from specified distributions. This probabilistic method is particularly advantageous in high-dimensional search spaces, where an exhaustive grid search would be computationally infeasible. By randomly selecting combinations, it can uncover promising configurations with fewer evaluations.

For Logistic Regression, the regularization parameter C  was sampled from a log-uniform range (e.g., [1e-4, 1e2]), which is standard practice for exploring values across several orders of magnitude.

GridSearchCV performs an exhaustive search over a manually specified set of hyperparameter values. Each possible combination is evaluated systematically. Although computationally expensive, it guarantees that all specified configurations are tested, making it suitable for models with smaller, well-defined search spaces.

#### Cross-Validation framework.

To ensure robust performance estimation during tuning, 3-fold cross-validation was employed throughout the optimization process. The training data was partitioned into three subsets, with each fold used once for validation while the remaining two were used for training. This strategy ensured consistent evaluation across different hyperparameter settings and helped mitigate overfitting.

While these search strategies were primarily applied to machine learning models, deep learning architectures were tuned manually using early stopping and learning rate scheduling due to their higher computational cost. For transparency and reproducibility, we report the post-optimization test accuracies obtained under RandomizedSearchCV and GridSearchCV (Table 5) using the same train–test protocol described above.

### Deep learning models

We implemented and evaluated a diverse suite of deep learning architectures using TensorFlow and Keras, chosen to explore various approaches to modeling complex, time-series meteorological data. All models were uniformly configured with the Adam optimizer and categorical_crossentropy loss function. Training efficiency and overfitting prevention were managed using EarlyStopping (patience = 10) and ReduceLROnPlateau (patience = 5) callbacks. For sequential models, input data was reshaped to a 3D format ([samples, timesteps = 1, features]) for compatibility.

#### Feedforward neural networks.

Initial exploration involved feedforward neural networks, which excel at capturing intricate non-linear relationships among static features. A standard **Artificial Neural Network (ANN)** served as a foundational baseline, comprising sequential dense layers (64, 32 units) with dropout (0.3) for regularization. To investigate the benefits of increased model capacity and training stability, a **deeper Neural Network (DNN)** was designed with more layers and integrated BatchNormalization. This DNN featured cascading dense layers (256, 128, 64 units), each followed by batch normalization, ReLU activation, and dropout (0.4 or 0.3).

#### Sequential Neural Networks.

Recognizing the temporal nature of weather data, we then deployed models specialized in processing sequential patterns. A **1D Convolutional Neural Network (CNN)** was utilized to automatically learn and extract local features across the feature dimension (e.g., interactions between different weather parameters). Its architecture included a Conv1D layer (64 filters, kernel size 3), followed by BatchNormalization, MaxPooling, and dense layers for classification.

For direct temporal modeling, Long Short-Term Memory (LSTM) Networks were employed, known for their ability to maintain memory states across sequences (100 units, then 50 units). To further enhance sequential understanding by processing data in both forward and backward directions, a **Bidirectional LSTM (Bi-LSTM) Network** was also implemented. This bidirectional approach allows the model to leverage context from both past and future observations, often leading to improved performance on time-series data.

This multi-faceted approach, leveraging both static and sequential deep learning architectures, allowed us to comprehensively assess how different architectural strengths address the inherent complexity and temporal dynamics of rainfall classification. To further clarify the rationale behind the inclusion of these models and their respective capabilities, the following summarizes their complementary strengths:

#### Model capability and rationale.

The selected models encompass complementary algorithmic capabilities suitable for the complex, non-linear, and temporally dependent nature of rainfall processes.

Ensemble-based machine learning models such as **Random Forest**, **Gradient Boosting**, **XGBoost**, and **LightGBM** are effective at capturing non-linear feature interactions and reducing variance through bagging and boosting, while maintaining interpretability via feature importance. **CatBoost** further improves handling of categorical features and class imbalance, making it well suited for heterogeneous meteorological datasets.

In contrast, deep learning architectures provide the ability to model temporal and sequential dependencies. The **ANN** and **DNN** capture complex multi-dimensional relationships among meteorological variables; the **1D-CNN** extracts local temporal patterns through convolutional filters; and the recurrent architectures (**LSTM** and **Bi-LSTM**) learn long and bidirectional dependencies in rainfall time series, effectively representing delayed and feedback-driven atmospheric dynamics.

Including both classical ensemble and deep sequential models enables a balanced comparison of static and dynamic predictive mechanisms, ensuring that the conclusions are not biased toward a single modeling family. Together, these methodological choices establish a diverse and balanced experimental framework capable of modeling both static and dynamic rainfall characteristics.

### Ensemble modeling strategies

To enhance predictive robustness and generalization, we employed several ensemble learning strategies that combine outputs from complementary model families. Ensemble learning aggregates the predictions of multiple base learners, reducing variance and bias while improving stability. In this study, we identify two ensembles as **primary approaches**, a soft-voting ensemble and a two-stage stacking framework, while additional methods (Mixture-of-Experts, Feature-View Ensembling, Dynamic Ensemble Selection, Cascade Generalization, and Greedy Selection) are reported as **ablation ensembles** for comparative insight.

#### Soft-Voting ensemble of top boosters.

Ensemble methods combine predictions from multiple base learners to reduce variance, mitigate individual model biases, and improve generalization performance. Among various ensemble strategies, soft voting aggregates the class probability estimates from each constituent classifier and assigns the final prediction to the class with the highest averaged probability. This probabilistic averaging approach is particularly advantageous for multi-class classification tasks because it leverages the calibrated confidence estimates of each model rather than relying solely on hard class labels.

The rationale for selecting a soft-voting ensemble in this study stems from the complementary strengths of different tree-based learning paradigms. Bagging-based methods such as Random Forest reduce variance by averaging predictions across independently trained decision trees on bootstrapped samples, making them robust to overfitting but potentially less adaptive to complex patterns. In contrast, boosting-based methods such as XGBoost and LightGBM sequentially construct trees that focus on correcting the residual errors of preceding learners, yielding high predictive accuracy but with increased sensitivity to noisy samples. By combining these complementary paradigms, the soft-voting ensemble exploits the variance-reduction properties of bagging while benefiting from the bias-reduction capabilities of boosting.

Specifically, the ensemble was constructed using three top-performing classifiers: Random Forest (representing bagging), LightGBM (gradient boosting with histogram-based leaf-wise growth), and XGBoost (regularized gradient boosting with level-wise growth). These models were selected based on their individual performance during preliminary experiments and their documented success on structured tabular data in meteorological and environmental applications. The ensemble was implemented using the VotingClassifier class from scikit-learn with voting = ’soft’.

The final predicted probability for each class is computed as the weighted average of the individual model probabilities [31], as shown in Equation 12:


$$\hat{P}(y=c|x)=\sum_{i=1}^{N}w_{i}\cdot P_{i}(y=c|x)\;$$ (12)

where $P_{i}(y=c|x$ denotes the probability of class *c* estimated by model *i* for input instance *x*, $w_{i}\;$ represents the weight assigned to model *i*, and N=3  is the number of ensemble members. The weights were tuned via grid search on the validation set to maximize the macro-averaged F1-score, ensuring balanced performance across all rainfall intensity classes including the minority “Very Heavy Rain” category.

The primary advantage of this soft-voting design is improved stability: even if one constituent model produces an erroneous prediction with moderate confidence, the ensemble can still yield the correct classification if the other models agree. Additionally, by averaging probability estimates, the ensemble tends to produce better-calibrated confidence scores than individual models—a property that directly supports the reliability analysis presented later in this study. The main limitation is the increased computational cost during inference, as predictions must be obtained from all three base models; however, this overhead is negligible for the deployment scenarios considered in this work.

#### Two-stage hierarchical stacking (primary ensemble).

**Fig 8:**
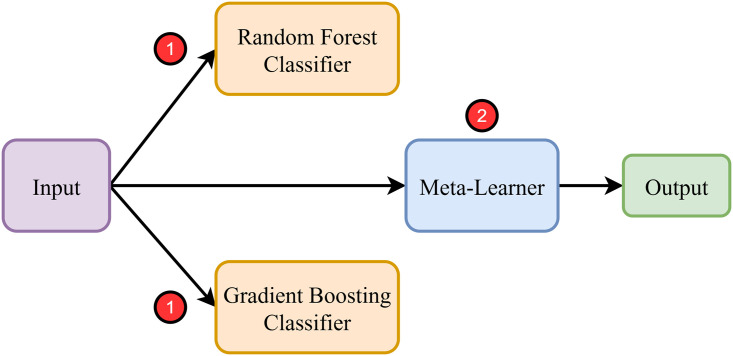
Two-stage hierarchical stacking framework. Stage 1 produces out-of-fold (OOF) predictions from base learners; these OOF probabilities form meta-features used by the Stage 2 meta-learner for final prediction.

Hierarchical stacking was adopted as the main ensemble protocol. In the first stage, diverse classifiers are trained on the original feature set using **Stratified 5-Fold Cross-Validation**. Out-of-fold (OOF) probabilities preserve class balance while preventing data leakage. These OOF predictions are concatenated to form meta-features for the second stage. The overall architecture of this two-stage stacking framework is illustrated in Fig 8.

- **Stage 1 (Base Learners):** Random Forest, LightGBM, XGBoost, CatBoost, Decision Tree, K-Nearest Neighbors, and Logistic Regression.
- **Stage 2 (Meta-Learner):** Random Forest trained on OOF meta-features.

Formally, for base learners $f_{1},f_{2},\ldots ,f_{k}\;$, the meta-feature vector is defined in Equation 13 [38]:


$$Z(x)=[\begin{array}{c} f_{1}(x)\\ f_{2}(x)\\ \vdots \\ f_{k}(x) \end{array}],\;\hat{y}=g(Z(x))\;$$ (13)

where g(·) is the meta-learner minimizing classification loss. All OOF predictions were generated exclusively within training folds; the independent test set was never used in training or model selection.

#### Mixture-of-Experts with gating network.

**Fig 9:**
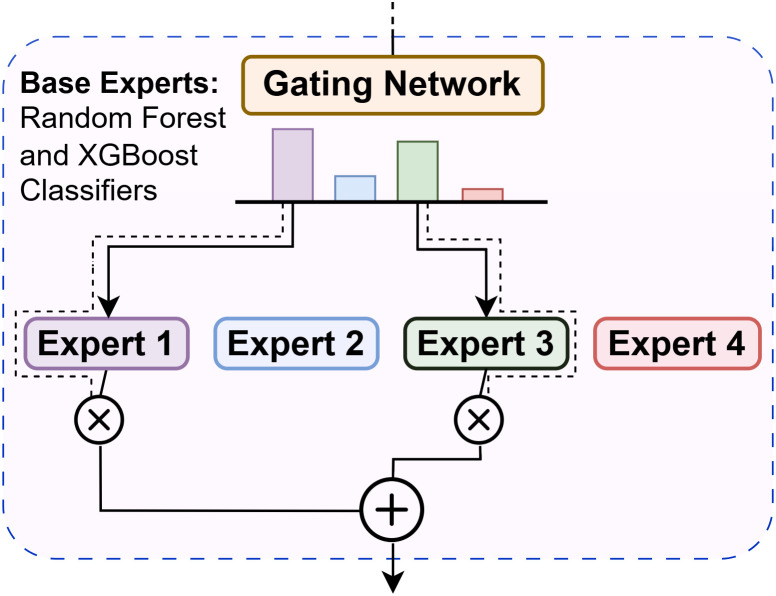
Mixture-of-experts (MoE) architecture. The figure illustrates how a gating network adaptively assigns weights to expert models (Random Forest and XGBoost) for each input sample.

The Mixture-of-Experts model (see Fig 9) dynamically assigns data instances to the most competent expert [39]. A gating neural network learns to produce a soft selection over experts using a softmax activation:


Gating Output=softmax(fc(x)).  (14)

The final prediction is a weighted sum of expert outputs based on the gating probabilities. Training followed the same 5-fold OOF protocol to avoid information leakage.

- **Base Experts:** Random Forest and XGBoost classifiers.
- **Gating Network:** A neural network that outputs softmax probabilities over experts.

#### Feature-View ensembling.

Feature-View Ensembling partitions input features into semantically distinct subsets, each modeled independently to capture domain-specific relationships. For this study, the features were grouped as follows:

- **Meteorological:** Sunshine, Humidity, Temperature
- **Temporal:** Month, Day (encoded cyclically as sine and cosine)
- **Location:** Station (label-encoded)

Each sub-model $f_{i}({𝐱}_{i})$ generated a probability vector ${\hat{p}}_{i}\;$, concatenated as:


$$𝐳=[{\hat{p}}_{\text{met}},{\hat{p}}_{\text{temp}},{\hat{p}}_{\text{loc}}],\;\hat{y}=g(𝐳),\;$$ (15)

where g(·) is a Random Forest meta-classifier trained on concatenated outputs. This setup allowed each model to specialize on its respective feature domain while maintaining interpretability.

#### Dynamic ensemble selection.

**Fig 10:**
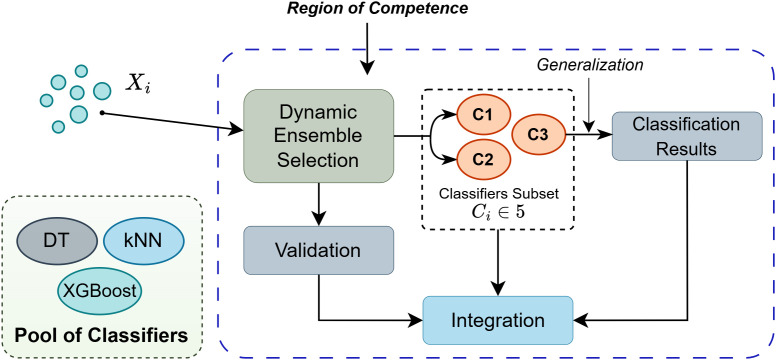
Dynamic ensemble selection (DES) using the KNORA-U algorithm. The figure illustrates how the KNORA-U algorithm selects classifiers (Decision Tree, Random Forest, KNN, and XGBoost) based on local neighborhood accuracy.

Dynamic Ensemble Selection (DES) adapts the chosen ensemble per test instance. Using the KNORA-U algorithm [40], classifiers with the highest local competence in the neighborhood of the test sample are retained for final voting. The competence region was estimated using a k-nearest neighbor search over validation data. This dynamic strategy ensures only locally competent classifiers contribute to the final decision, reducing noise from less reliable models. The overall process is illustrated in Fig 10.

#### Cascade generalization.

Cascade Generalization, also known as deep stacking, iteratively refines predictions across multiple layers [41]. Each layer receives both the original features and the predictions from the preceding layer, enabling each successive stage to model residual errors and inter-feature dependencies.


$${𝐗}_{1}={𝐗}_{\text{train}},\;{𝐗}_{k}=[{𝐗}_{\text{train}}\;||\;P_{k-1}({𝐗}_{k-1})],\;$$ (16)

$$P_{k}=f_{k}({𝐗}_{k}),\;\hat{y}=P_{K}.\;$$ (17)

This progressive integration enables higher layers to model residual errors and dependencies unaccounted for by earlier learners.

#### Greedy ensemble selection.

Greedy Ensemble Selection incrementally constructs an ensemble by adding models that yield the largest gain in validation accuracy [42]:


$$M^{*}=\arg\max_{M\in ℳ⧵{𝒮}_{t-1}}\text{Acc}({𝒮}_{t-1}\cup \{M\},{𝒟}_{val}).\;$$ (18)

If accuracy improves, $M^{*}\;$ is retained; otherwise, selection stops. The candidate pool comprised Random Forest, Gradient Boosting, XGBoost, and Logistic Regression. Notably, the selection process converged to a singleton ensemble (Random Forest), reaffirming its dominant predictive capacity within the model pool.

All ablation ensembles were trained under identical Stratified 5-Fold OOF splits; the independent 20% test set was reserved exclusively for final evaluation. This ensured that performance comparisons across ensembles remained fair and unbiased.

## Results and discussion

### Experimental setup

The experiments were conducted using a combination of cloud-based and local computing resources. We utilized Google Colab and Kaggle for its accessibility and pre-configured machine learning libraries. Local experimentation and model training were performed on a personal computer equipped with an Intel Core i5 processor and 16 GB of RAM. This setup allowed for flexible development, evaluation, and deployment of our rainfall classification models.

The following sections detail the performance evaluation of several machine learning models applied to the task of rainfall prediction. The experiments were conducted using different versions of the dataset: the original, preprocessed (with outlier removal), and preprocessed with SMOTE applied. For each model and setting, standard classification metrics, including training and testing accuracy, precision, recall, and F1-score, were computed and are summarized in the accompanying tables. All tables are formatted to fit within a single column of a two-column document layout for consistency and readability.

#### Real-world reliability evaluation.

**Fig 11:**
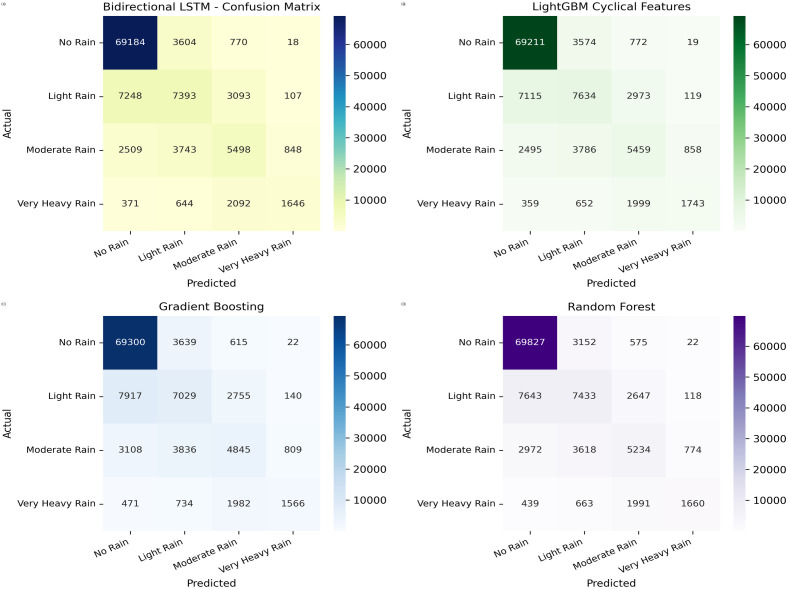
Confusion matrices of the top-performing models. The figure presents results for (a) Bidirectional LSTM, (b) LightGBM (cyclical features), (c) Gradient Boosting, and (d) Random Forest.

To emulate real-world deployment, all models were trained on 80% of the data and evaluated on a completely held-out 20% test set with no record overlap. Lag and rolling features were computed per station using only historical observations, and the original Rainfall column was removed after creating Rainfall_Class to avoid target leakage. Cross-validation was employed during hyperparameter tuning to confirm stability across folds. Reliability was further assessed on the independent test set through class-wise Precision, Recall, and F1-scores, along with confusion matrices (Fig 11; Table 3) which reflect operational performance in detecting both frequent (No Rain) and minority (Very Heavy Rain) events. SMOTE was applied only as an ablation on the training data and was excluded from final models due to degraded test performance, a conservative choice that preserves temporal coherence and the empirical data distribution.

**Table 3:** Performance comparison of ML and DL models with and without feature engineering.

	Without Feature Engineering				With Feature Engineering			
Model	Acc	Prec	Recall	F1	Acc	Prec	Recall	F1
*Machine Learning Models*								
Decision Tree	0.671	0.68	0.67	0.67	0.703	0.71	0.70	0.70
Random Forest	**0.740**	0.70	0.74	0.71	**0.774**	0.75	0.77	0.76
Logistic Regression	0.710	0.65	0.71	0.65	0.736	0.70	0.74	0.70
Gradient Boosting	0.720	0.69	0.72	0.70	0.761	0.74	0.76	0.75
KNN	0.710	0.67	0.71	0.69	0.721	0.69	0.72	0.70
AdaBoost	0.710	0.68	0.71	0.69	0.735	0.71	0.74	0.72
Naive Bayes	0.710	0.68	0.71	0.69	0.607	0.71	0.61	0.64
XGBoost	0.710	0.68	0.71	0.69	0.773	0.75	0.76	0.76
LightGBM	0.730	0.70	0.73	0.71	0.773	0.76	0.77	0.76
CatBoost	0.660	0.73	0.66	0.68	0.769	0.75	0.76	0.75
*Deep Learning Models*								
ANN	**0.724**	0.54	0.41	0.44	0.764	0.61	0.51	0.54
DNN	0.723	0.55	0.40	0.42	0.767	0.61	0.52	0.55
1D CNN	0.700	0.50	0.32	0.31	0.767	0.62	0.52	0.55
LSTM	0.676	0.17	0.25	0.20	0.769	0.62	0.52	0.56
Bi-LSTM	0.676	0.17	0.25	0.20	**0.770**	0.62	0.53	0.56

### Evaluation metrics

To evaluate model performance, we employed four standard classification metrics. Let *TP*, *FP*, *FN*, and *TN* denote the number of true positives, false positives, false negatives, and true negatives, respectively.

• **Accuracy:** Proportion of correctly classified samples among all samples:


$$\text{Accuracy}=\frac{TP+TN}{TP+TN+FP+FN}\;$$

• **Precision:** Fraction of correctly predicted positive instances among all predicted positives:


$$\text{Precision}=\frac{TP}{TP+FP}\;$$

• **Recall (Sensitivity):** Fraction of correctly predicted positive instances among all actual positives:


$$\text{Recall}=\frac{TP}{TP+FN}\;$$

• **F1-score:** Harmonic mean of precision and recall, balancing false positives and false negatives:


$$\text{F1}=2\cdot \frac{\text{Precision}\cdot \text{Recall}}{\text{Precision}+\text{Recall}}\;$$

For the multi-class setting, we report **macro-averaged Precision, Recall, and F1-score**, where the metric is first computed independently for each class and then averaged across classes with equal weight. This averaging prevents dominance by the majority “No Rain” class. Additionally, confusion matrices were generated for selected models to provide class-wise insight into misclassification patterns.

### Baseline model performance

Table 3 summarizes the performance of all machine learning and deep learning models on two settings: (i) the baseline dataset with raw features, and (ii) the enhanced dataset after applying feature engineering. This comparison highlights the incremental improvements gained from incorporating lag, rolling, and cyclical temporal features, in contrast to the baseline performance on unprocessed inputs.

Among all models, Random Forest achieved the highest test accuracy of 0.74, demonstrating strong generalization. Decision Tree significantly overfitted the training data, achieving perfect accuracy on the training set but underperforming on the test set. Ensemble models like LightGBM and CatBoost also showed competitive performance with relatively balanced precision and recall.

**Fig 12:**
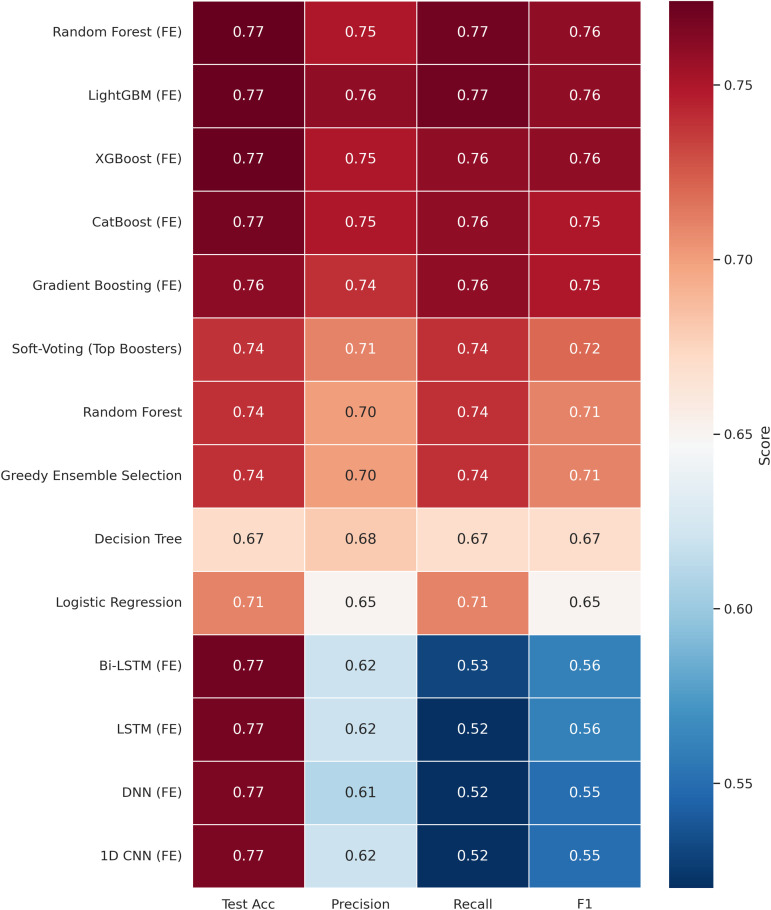
Unified multi-metric heatmap summarizing model performance. The figure presents accuracy, precision, recall, and F1-score across all model categories (baseline, preprocessed, feature-engineered, and ensemble). Darker tones indicate stronger performance and highlight the relative consistency between models.

As shown in Fig 12, baseline models exhibit closely grouped performance across all evaluated metrics, reflecting stable initial behavior before feature-engineering transformations. This stability provides a clear benchmark for assessing the incremental gains achieved through preprocessing and ensemble integration. The comparative visualization approach was inspired by the evaluation style of Ben Yahia *et al.* [43], which emphasizes clarity and interpretability in multi-metric model assessment.

**Fig 13:**
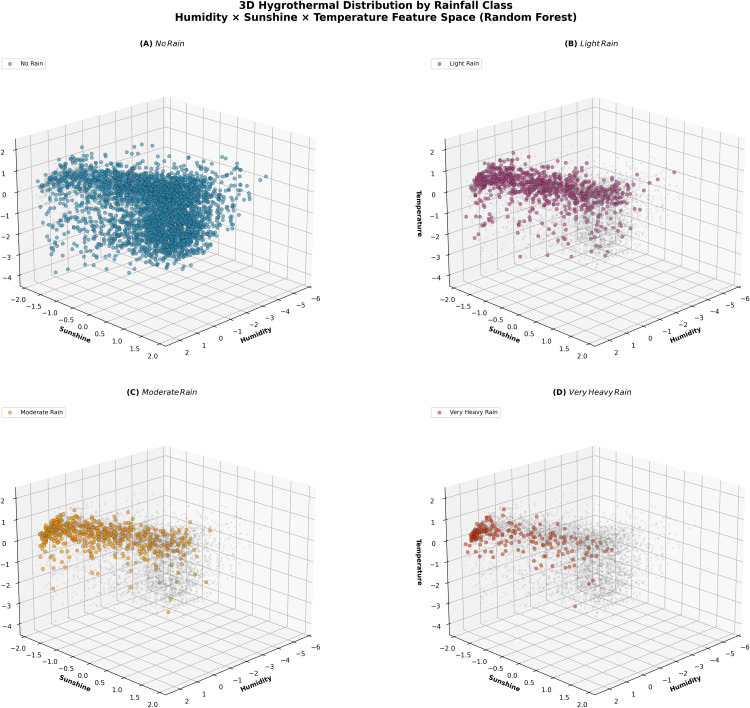
Three-dimensional probability distributions of rainfall predictions. The figure shows rainfall classification probabilities across humidity, sunshine, and temperature feature spaces using the Random Forest model. Color gradients indicate classification confidence, revealing how hygrothermal variables jointly influence rainfall occurrence.

To further interpret the model‘s hygrothermal dynamics, a three-dimensional probability distribution analysis (Fig 13) was performed across humidity, sunshine duration, and temperature. The visualization exposes distinct confidence gradients, where high humidity coupled with limited sunshine corresponds to increased rainfall likelihood, whereas dry, high-radiation conditions are associated with near-zero probabilities. This representation follows the probabilistic decision surface methodology proposed by Ben Yahia *et al.* [44], originally applied to classify hygrothermal comfort zones in Mediterranean climates. By analogy, our rainfall probability mapping extends this framework to atmospheric classification tasks, demonstrating that humidity and sunshine duration remain the key boundary-defining variables shaping model confidence.

### Impact of data balancing on model performance

In Table 4, SMOTE was applied to address class imbalance after standard preprocessing. The goal was to enhance model generalization by providing a more balanced dataset during training.

**Table 4:** Model performance after data balancing using SMOTE.

Model	Test Acc	Precision	Recall	F1
Decision Tree	0.261	0.60	0.26	0.29
Random Forest	0.380	0.71	0.38	0.43
Logistic Regression	0.420	0.69	0.42	0.49
Gradient Boosting	0.420	0.72	0.42	0.48
KNN	0.420	0.68	0.42	0.48
AdaBoost	0.430	0.70	0.43	0.49
Naive Bayes	0.430	0.70	0.43	0.49
XGBoost	0.210	0.70	0.21	0.21
LightGBM	**0.720**	0.69	0.72	0.70
CatBoost	0.650	0.72	0.65	0.68

Contrary to expectations, applying SMOTE led to a sharp decline in performance for most models, especially Decision Tree, Random Forest, and XGBoost. This suggests that the synthetic samples generated by SMOTE did not accurately reflect the underlying data distribution. However, LightGBM and CatBoost remained resilient and performed consistently across all experiments, indicating their robustness to class imbalance and synthetic data artifacts.

We attribute this behavior to a combination of algorithmic and data-related factors. First, SMOTE interpolates minority samples in feature space; with engineered temporal lags/rolling statistics and station-specific patterns, interpolation can yield synthetic feature vectors that are not physically realizable (e.g., inconsistent lag relationships) even when applied to the training set only. Second, extreme-rainfall days (minority classes) may be *multi-modal* (different meteorological regimes), so local interpolation can blur class boundaries and introduce ambiguous samples that increase confusion at test time. Third, when rare events contain noise or measurement artifacts, SMOTE can effectively amplify those artifacts by creating additional synthetic neighbors around them. Together, these effects can distort the empirical distribution that tree-based learners exploit, helping explain why cost-sensitive learning via class weighting generalized more reliably than oversampling in our experiments.

### Model performance after hyperparameter tuning

Following the baseline, preprocessing, and SMOTE experiments, we applied *RandomizedSearchCV* and *GridSearchCV* to probe the sensitivity of each algorithm to key hyperparameters (e.g., tree depth, number of estimators, learning rate, regularization, neighborhood size). The goal was to calibrate models for generalization rather than to overfit a particular split. Table 5 reports, for each classical model, the best test accuracy observed under the two search strategies.

**Table 5:** Test accuracies of classical models after hyperparameter optimization using Randomized Search (RSCV) and Grid Search (GSCV).

Model	RSCV Test Accuracy	GSCV Test Accuracy
Decision Tree	0.720	0.720
Logistic Regression	0.704	0.704
Gradient Boosting	0.722	0.720
AdaBoost	0.722	0.707
Random Forest	0.728	**0.730**
XGBoost	0.718	0.718
KNN	0.731	0.721

Across algorithms, tuned test accuracies were *comparable* to the corresponding baselines (typically within ≤1–2 percentage points). For example, Random Forest attained 0.730 under GSCV, while its untuned baseline was 0.740, differences that fall within the variability observed across folds. This consistency suggests that performance is driven primarily by feature design (lag/rolling/cyclical encodings) rather than aggressive parameter search. In practice, tuning was most useful for stabilizing calibration and fold-to-fold behavior, which we leverage in the ensemble experiments that follow.

### Ensemble and Stacking Model Evaluation

Table 6 presents the performance of various ensemble stacking strategies applied to improve rainfall classification accuracy.

**Table 6:** Comparative performance of ensemble and stacking strategies.

Model Name	Test Accuracy	Precision	Recall	F1-Score
Soft-Voting of Top Boosters	0.739	0.71	0.74	0.72
Flat Stacking of All Models	0.734	0.70	0.73	0.71
Two-Stage Hierarchical Stacking	0.739	0.71	0.74	0.72
Dynamic Local Voting (DES)	0.732	0.70	0.73	0.71
Cascade Generalization (Deep Stacking)	0.732	0.71	0.73	0.72
Mixture-of-Experts with Gating Network	0.735	0.70	0.74	0.71
Feature-View Ensembling	0.680	0.66	0.68	0.67
Greedy Ensemble Selection (RandomForestClassifier)	**0.740**	0.70	0.74	0.71

Overall, the ensemble techniques provided performance that was comparable to or slightly better than individual base models.Among the balanced approaches, **Soft-Voting of Top Boosters** achieved strong performance with a test accuracy of 0.739 and an F1-score of 0.72.

The **Greedy Ensemble Selection** converged to a singleton ensemble RandomForestClassifier yielding a test accuracy of 0.740 and an F1-score of 0.71. In contrast, **Feature-View Ensembling** underperformed, suggesting that model diversity alone may not guarantee improved performance. These findings support the effectiveness of certain stacking strategies, especially when models are selected or combined with performance-aware mechanisms.

In this setting, a high-capacity Random Forest trained on lag, rolling, and cyclical features captured the dominant non-linear relationships within the data, leaving limited headroom for ensemble aggregation to yield further gains. Additionally, calibration mismatches among base learners and partial overlap in their learned decision boundaries likely reduced the effectiveness of probability-level combination.

### Deep learning model evaluation

To further explore the predictive capabilities for rainfall classification, we extended our analysis to include various deep learning architectures. Deep learning models are particularly well-suited for capturing complex, non-linear relationships and temporal dependencies in large datasets, which are characteristic of meteorological phenomena. We evaluated Artificial Neural Networks (ANN), Deep Neural Networks (DNN), 1D Convolutional Neural Networks (CNN), Long Short-Term Memory (LSTM) networks, and Bidirectional LSTM (Bi-LSTM) networks. Table 3 reports the baseline performance of the deep learning models when trained on the raw dataset without feature engineering, which serves as a reference point for evaluating the gains achieved with engineered features.

The deep learning models generally showed limited performance, especially in detecting minority classes. ANN and DNN achieved moderate accuracies around 0.72, but their macro-averaged F1-scores were low (0.44 and 0.42, respectively), indicating poor recall for rainfall events. The 1D CNN performed slightly worse, and both LSTM and Bi-LSTM models struggled significantly, effectively failing to classify any non-“No Rain” instances, resulting in very low macro-averaged metrics. This suggests that these models, despite their capacity for learning complex patterns, struggled with the raw sequential data and high class imbalance without explicit feature guidance.

### Effect of feature engineering on predictive performance

The performance of various machine learning models, evaluated on Feature Set 1 as described in Section 3.3, is presented in Table 3. The table lists the accuracy, macro-averaged precision, macro-averaged recall, and macro-averaged F1-score for each model. Accuracy reflects the overall correctness of predictions, while the macro-averaged metrics provide a balanced measure of precision, recall, and F1-score across all classes, accounting for class imbalance. The best values in each column are highlighted in bold. The inclusion of engineered features markedly improved model performance across both traditional machine learning and deep learning models (Table 3), over baseline methods that utilized raw or minimally processed data. Notably, top-performing models like Random Forest and LightGBM achieved accuracies around **77%**, while several deep learning models (DNN, 1D CNN, LSTM, Bi-LSTM) also reached similar peak accuracies of approximately 77% after feature engineering. This enhancement is primarily due to the use of lag and rolling window features, which effectively capture temporal dependencies and trends within the data. We observed that the ‘Humidity_Lag_1‘ feature consistently exhibited the highest importance across all evaluated models. Furthermore, the cyclical encoding applied in Feature Set 2 adeptly represents seasonal patterns, enabling models to discern and leverage underlying temporal dynamics more effectively. To visually demonstrate these improvements, Fig 11 shows the confusion matrix of the best performing models.

### Model interpretability using LIME and SHAP

#### Local Explanations with LIME.

To make the rainfall classification model more transparent and interpretable, we used **LIME** (Local Interpretable Model-agnostic Explanations). LIME provides instance-level explanations by identifying the most influential features for a specific prediction.

**Fig 14:**
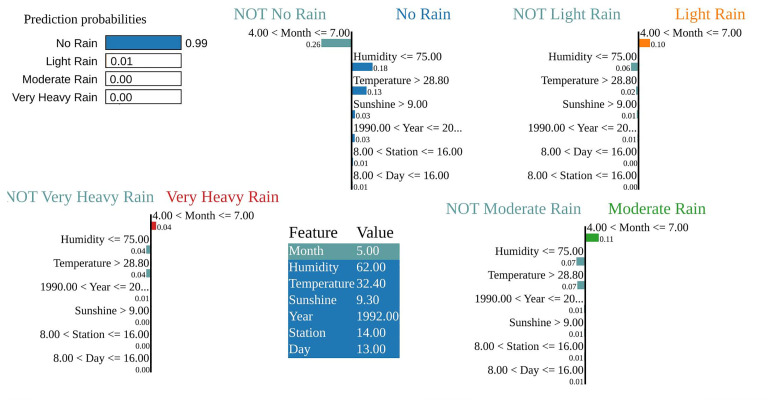
LIME visualization depicting local feature contributions for dry weather predictions. The figure illustrates how the LIME algorithm identifies the most influential features responsible for predicting ‘no rain‘ (dry weather) conditions.

In the visualized result, LIME is applied to a test instance classified as *“No Rain”* with a high confidence of **0.99**. Other class probabilities were very low (*Light Rain:* 0.01, *Moderate Rain:* 0.00, *Very Heavy Rain:* 0.00), as shown in Fig 14. The key contributing features for this classification were:

- Humidity ≤ 75.00 (contribution: + 0.18)
- Temperature > 28.80^°^C (contribution: + 0.13)
- Sunshine > 9.00 hours (contribution: + 0.03)

These factors represent typical dry-weather conditions—low humidity, high temperature, and high sunshine duration, which align well with the “No Rain” classification. The instance‘s meteorological profile (Humidity: 62.00, Temperature: 32.40^°^C, Sunshine: 9.30 hours) further supports this interpretation. Thus, LIME validates that the model‘s individual decisions are consistent with established domain knowledge.

#### Interpretation of Lagged and Rolling Variables.

In addition to same-day meteorological inputs, the model incorporates lagged and rolling mean features to capture short-term temporal dependencies. *Humidity_lag_1* denotes the humidity level from the previous day, reflecting short-term atmospheric moisture persistence. *Temperature_lag_3* corresponds to the temperature recorded three days earlier, capturing delayed thermal effects, while *Temperature_roll_mean_7* and *Humidity_roll_mean_3* represent seven-day and three-day moving averages that smooth short-term fluctuations.

#### Global Feature Contributions with SHAP.

To complement LIME‘s local interpretability, we employed **SHAP** (SHapley Additive exPlanations) for a global understanding of feature influence. SHAP quantifies each feature‘s contribution to the model output based on cooperative game theory, providing a consistent, dataset-wide interpretation.

**Fig 15:**
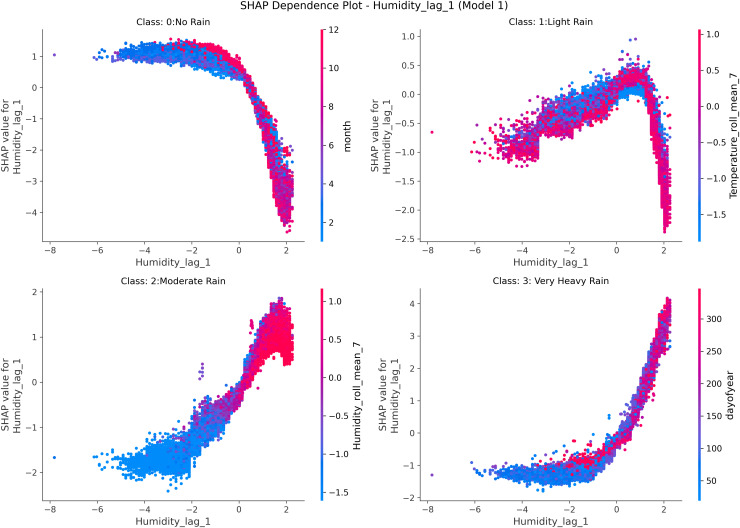
SHAP dependence plots for previous-day humidity across rainfall classes. The figure shows SHAP dependence plots for *Humidity_lag_1* (previous-day humidity) across rainfall classes: *no rain*, *light rain*, *moderate rain*, and *very heavy rain*. Color gradients indicate interactions with *Temperature_lag_3* (three-day lag), *Temperature_roll_mean_7* (seven-day rolling mean), and *Humidity_roll_mean_3* (three-day rolling mean). Higher lagged humidity values correspond to stronger SHAP contributions for light and very heavy rainfall, highlighting how recent temperature and humidity persistence jointly influence precipitation.

Fig 15 shows SHAP dependence plots for *previous-day humidity (Humidity_lag_1)* across rainfall classes: *No Rain*, *Light Rain*, *Moderate Rain*, and *Very Heavy Rain*. Higher lagged humidity values lead to increasingly positive SHAP values for the *Light Rain* and *Very Heavy Rain* classes, indicating a greater likelihood of precipitation. Conversely, lower humidity levels correspond to negative SHAP contributions, reinforcing predictions of dry weather.

Color gradients depict interactions with other variables such as *Temperature_lag_3*, *Temperature_roll_mean_7*, and *Humidity_roll_mean_3*. These interactions reveal how previous-day humidity, combined with recent temperature and humidity trends, influences rainfall probability, consistent with meteorological processes governing moisture buildup and convection.

The visualization layout was inspired by the probability distribution representations of Ben Yahia *et al.* [44], whose depiction of hygrothermal comfort zones guided the design of our feature-dependence interpretation. Together, LIME and SHAP provide complementary perspectives: LIME elucidates model reasoning for individual predictions, while SHAP validates that the learned relationships are physically consistent at the global scale.

### Reliability analysis and environmental decision support

Beyond standard classification metrics, we conducted post-processing analyses to assess prediction reliability and uncertainty quantification. These analyses address the practical requirement of transforming raw model outputs into actionable decision-support metrics for environmental applications.

#### Model calibration analysis.

Model calibration quantifies the alignment between predicted confidence scores and observed accuracy. A well-calibrated classifier produces probability estimates that accurately reflect the true likelihood of correct classification. We assessed calibration using reliability diagrams and the Expected Calibration Error (ECE):


$$\text{ECE}=\sum_{b=1}^{B}\frac{n_{b}}{N}\left|\text{acc}(b)-\text{conf}(b)\right|\;$$ (19)

where *B* denotes the number of confidence bins, $n_{b}\;$ is the sample count in bin *b*, and acc(b) and conf(b) represent the observed accuracy and mean confidence within each bin, respectively.

**Fig 16:**
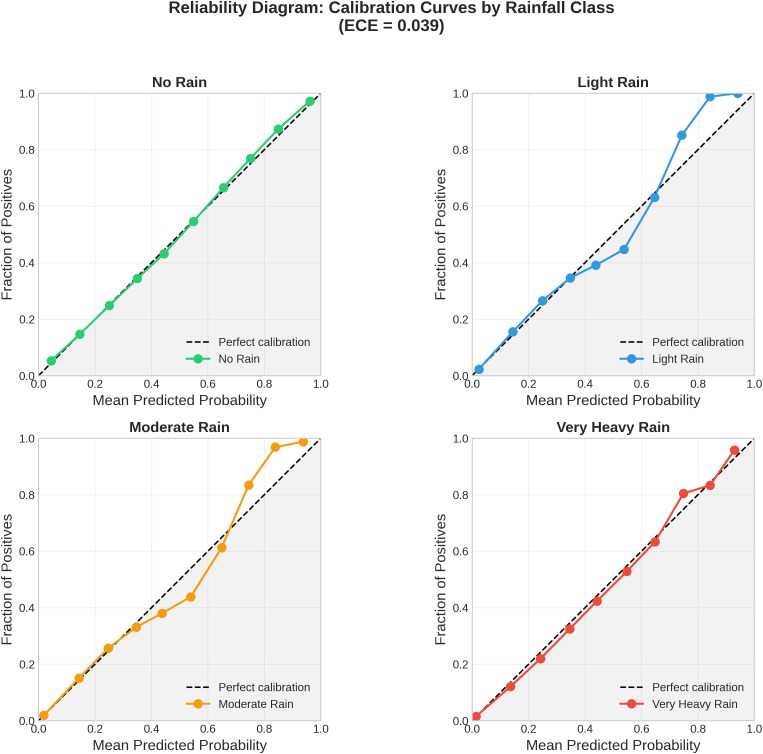
Reliability diagrams for rainfall intensity classification. Calibration curves show the relationship between mean predicted probability and fraction of positive outcomes for each rainfall class. The diagonal dashed line represents perfect calibration. The model achieves ECE = 0.039, demonstrating well-calibrated probabilistic predictions.

The Random Forest model achieved an ECE of 0.039, indicating excellent calibration suitable for probabilistic decision-making. Fig 16 presents class-wise reliability diagrams showing close alignment between predicted probabilities and observed frequencies. The No Rain class exhibits near-perfect calibration, closely tracking the diagonal reference line. Light Rain and Moderate Rain display slight over-confidence at higher probability ranges, while Very Heavy Rain maintains reasonable calibration despite its low base rate in the dataset.

#### Confidence-stratified accuracy analysis.

**Fig 17:**
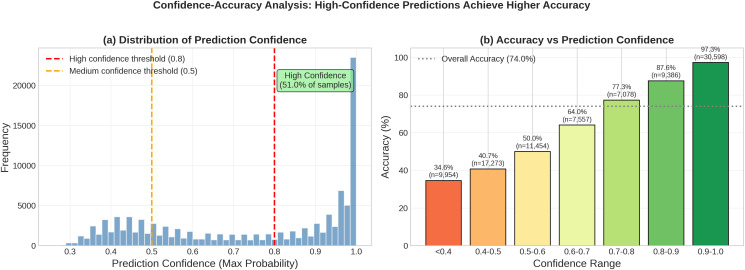
Confidence-accuracy analysis. **(a)** Distribution of prediction confidence scores; 42.0% of samples exceed the high-confidence threshold of 0.8. **(b)** Accuracy stratified by confidence level, showing that predictions with confidence 0.9–1.0 achieve 98.9% accuracy compared to the baseline accuracy of 70.1%.

We further examined the relationship between prediction confidence (maximum class probability) and classification accuracy (Fig 17). This analysis reveals a strong monotonic relationship spanning from near-random performance at low confidence to near-perfect accuracy at high confidence.

Predictions in the 0.9–1.0 confidence range achieve 98.9% accuracy (n = 36,130), while those in the 0.8–0.9 range achieve 93.7% accuracy (n = 9,267). Collectively, 42% of test samples exceed the 0.8 confidence threshold, representing a substantial subset suitable for high-reliability automated decision-making. The strong monotonic trend—ranging from 33.2% accuracy at low confidence (< 0.4) to 98.9% at high confidence (>0.9)—validates that the model’s confidence estimates reliably indicate prediction quality. This property enables practitioners to selectively deploy high-confidence predictions while flagging uncertain cases for human review.

### Implications for environmental decision support

The reliability analysis enables a confidence-aware operational framework with direct applications to environmental management:

- **Flood early warning:** High-confidence extreme rainfall predictions can trigger proactive alerts for flood-prone deltaic regions, enabling timely evacuation and resource mobilization.
- **Agricultural planning:** The high-confidence subset (98.9% accuracy) provides reliable guidance for irrigation scheduling and crop management with minimal false alerts.
- **Tiered automation:** The 42% of predictions exceeding the 0.8 confidence threshold can be deployed in fully automated monitoring systems, while lower-confidence predictions are routed for human review, optimizing resource allocation.
- **Uncertainty communication:** Confidence scores can be communicated alongside predictions, enabling stakeholders to calibrate responses according to forecast reliability.

This reliability-aware framework transforms standard classification outputs into decision-grade probabilistic forecasts, addressing the operational requirements of environmental monitoring and disaster preparedness in Bangladesh’s monsoon climate.

## Discussion

Our study presents a comprehensive and interpretable framework for rainfall intensity classification in Bangladesh, effectively modeling the nonlinear and temporal dependencies of monsoon rainfall. The top-performing models Random Forest (77.37%), LightGBM (77.27%), and Bi-LSTM (76.97%) demonstrate the complementary strengths of ensemble and sequence-based learning paradigms. Advanced temporal feature engineering, including lag, rolling, and cyclical encodings, enhanced temporal awareness and improved model accuracy by approximately 4%, with *Humidity_Lag_1* identified as the most influential predictor. Interpretability analyses using both LIME and SHAP confirmed that humidity, sunshine, and temperature are the dominant factors influencing rainfall intensity, aligning model behavior with established meteorological principles and ensuring trustworthiness in policy and decision-making contexts. SHAP dependence plots further revealed strong interactions between lagged humidity and temperature, providing physically consistent global explanations of model predictions. Despite efforts to mitigate class imbalance, SMOTE reduced model performance by distorting natural rainfall distributions, indicating that future research should explore cost-sensitive learning. Ensemble strategies such as soft voting and greedy selection improved overall robustness and stability. As highlighted in Table 7, the proposed framework represents one of the most comprehensive rainfall classification efforts in Bangladesh, utilizing a substantially larger and more diverse dataset than previous studies and providing a unified nationwide perspective on rainfall modeling. Overall, this work combines accuracy, interpretability, and scalability offering a practical foundation for climate aware decision support across agriculture, hydrology, and disaster management sectors.

**Table 7:** Comparative overview of recent rainfall prediction and classification studies.

Study	Study Area / Scale	Dataset and Objective	Modeling Approach	Main Findings / Performance	Remarks and Key Differences
Billah et al. (2022) [3]	Bangladesh (single station)	Daily rainfall prediction (regression) using historical data	LSTM compared with classical ML models	LSTM achieved ∼97% accuracy on a small dataset (∼2,300 samples)	Single-station regression; no intensity classification or ensemble approach.
Ria et al. (2021) [11]	Bangladesh (limited coverage)	Rainfall prediction and classification using local meteorological data	Random Forest, Decision Tree, SVM, KNN	RF achieved highest performance (exact metric not reported)	Small dataset (< 2.5k samples); limited feature and temporal design.
Di Nunno et al. (2022) [45]	Northern Bangladesh (regional)	Monthly precipitation forecasting (continuous regression)	M5P, SVR, hybrid M5P–SVR	Hybrid model reached $R^{2}\;$ up to 0.90 (Sylhet station)	Regional focus; regression only; no discrete rainfall classes or interpretability.
Rajab et al. (2023) [1]	Bangladesh (flood-linked)	Rainfall and flood forecasting using climatic indicators	Polynomial Regression, Random Forest, LSTM	RF $R^{2}\approx 0.76\;$; LSTM loss ∼0.09	Flood-focused; fewer stations; different prediction target.
Wani et al. (2024) [20]	NW Himalayas (India)	Rainfall prediction across altitudinal gradients and stations	ML, DL, and time-series models	DL models outperformed ML (lower RMSE, MAE)	Different geography and climate; complementary demonstration of ML/DL efficacy.
**Ours**	**Bangladesh (35 stations, nationwide)**	**543,839 daily samples; 4-class rainfall intensity classification**	**Integrated ML & DL ensembles (stacking, MoE, DES) with SHAP/LIME**	**RF: 77.4%; Bi-LSTM: 77.0%; stable macro-F1 across all classes**	**Largest unified daily dataset; advanced temporal features; interpretable ensemble framework with hydrological insight.**

## Conclusions

This research develops a large-scale, interpretable framework for rainfall intensity classification in Bangladesh, leveraging a comprehensive dataset of over 543,000 daily weather records from 35 stations nationwide. By integrating ensemble-based and deep learning models with advanced temporal feature engineering, the framework effectively captures the nonlinear and seasonal characteristics of monsoon rainfall. Random Forest and Bi-LSTM models emerged as the most effective, highlighting the complementary advantages of ensemble diversity and sequential modeling. Beyond achieving strong predictive performance, the framework emphasizes transparency and explainability through both LIME and SHAP analyses. These methods consistently identified humidity, sunshine duration, and temperature as key determinants of rainfall, offering meteorologically meaningful insights that strengthen confidence in model-driven decision-making. Additionally, the study demonstrates that conventional oversampling methods like SMOTE may distort meteorological patterns, emphasizing the need for domain-aware approaches to handle data imbalance. Nevertheless, certain limitations persist. The daily temporal resolution constrains real-time flood forecasting, while class imbalance and spatial interdependencies across stations remain challenges for model generalization. Future research should focus on integrating spatially aware deep learning models, such as graph-based or attention-driven architectures, and extending interpretability analyses to a global scale to better understand regional rainfall dynamics. In summary, this work establishes a scalable and interpretable framework for nationwide rainfall classification, contributing to climate-resilient decision support systems in Bangladesh. It sets the foundation for future advancements leveraging higher-resolution data, spatial-temporal modeling, and adaptive ensemble learning to further improve meteorological prediction and preparedness.
